# A Local Neighborhood Robust Fuzzy Clustering Image Segmentation Algorithm Based on an Adaptive Feature Selection Gaussian Mixture Model

**DOI:** 10.3390/s20082391

**Published:** 2020-04-22

**Authors:** Hang Ren, Taotao Hu

**Affiliations:** 1 Changchun Institute of Optics, Fine Mechanics and Physics, Chinese Academy of Sciences, Changchun 130033, China; renhang10@163.com; 2 Key Laboratory of Airborne Optical Imaging and Measurement, Changchun Institute of Optics, Fine Mechanics and Physics, Chinese Academy of Sciences, Changchun 130033, China; 3 School of Physics, Northeast Normal University, Changchun 130024, China

**Keywords:** fuzzy clustering, image segmentation, feature selection, neighborhood information

## Abstract

Since the fuzzy local information C-means (FLICM) segmentation algorithm cannot take into account the impact of different features on clustering segmentation results, a local fuzzy clustering segmentation algorithm based on a feature selection Gaussian mixture model was proposed. First, the constraints of the membership degree on the spatial distance were added to the local information function. Second, the feature saliency was introduced into the objective function. By using the Lagrange multiplier method, the optimal expression of the objective function was solved. Neighborhood weighting information was added to the iteration expression of the classification membership degree to obtain a local feature selection based on feature selection. Each of the improved FLICM algorithm, the fuzzy C-means with spatial constraints (FCM_S) algorithm, and the original FLICM algorithm were then used to cluster and segment the interference images of Gaussian noise, salt-and-pepper noise, multiplicative noise, and mixed noise. The performances of the peak signal-to-noise ratio and error rate of the segmentation results were compared with each other. At the same time, the iteration time and number of iterations used to converge the objective function of the algorithm were compared. In summary, the improved algorithm significantly improved the ability of image noise suppression under strong noise interference, improved the efficiency of operation, facilitated remote sensing image capture under strong noise interference, and promoted the development of a robust anti-noise fuzzy clustering algorithm.

## 1. Introduction

### 1.1. Image Segmentation Algorithms

Existing image segmentation methods are mainly divided into the following categories: the edge-based image segmentation methods, the region-based image segmentation methods, and the image segmentation methods based on a specific theory. Cluster segmentation, as a typical unsupervised segmentation method, has attracted the attention of many scholars and has been widely used and studied in many fields [1,2].

Clustering algorithms can be divided into hard partition clustering algorithms and soft partition clustering algorithms. Hard partition clustering algorithms are used for image segmentation. Their principle is to directly divide an image according to the similarity of pixels in terms of qualities such as grayness, color, and texture. The optimal solution or partition can be obtained by minimizing the objective function for algorithms such as the H-means algorithm [3,4], the global K-means algorithm, and the K-means algorithms, where K-means clustering is one of these methods [5,6]. This clustering algorithm has the advantages of fast segmentation, clear structure, and good usability [7,8], but it is also prone to fall into local minima in the process of optimizing the segmentations. Soft partitioning clustering algorithms use the degree of belonging or the probability of pixels to indirectly partition the similarity of pixels and search for an optimal decomposition in the process of minimizing the likelihood function of the objective function or maximizing the parameter [9,10]. For example, Dunn [4] proposed the fuzzy C-means clustering algorithm in 1947. In 1981, Bezdek [5] proved and compared the measurement theory of mean clustering and fuzzy mean clustering, and proved the convergence of the fuzzy mean clustering algorithm, established fuzzy clustering theory, promoted the development of the fuzzy clustering algorithm, and developed the fuzzy mean clustering algorithm such that it became an important branch of fuzzy theory. The theory was introduced into the clustering algorithm to improve the adaptability of the algorithm, which has been widely used [11,12].

### 1.2. Fuzzy Clustering Algorithm Based on Feature Selection

At present, the research of clustering analysis focuses on the scalability of the clustering method, the validity of clustering for complex shapes and data types, high-dimensional clustering analysis technology, and the clustering method for mixed data. Among them, high-dimensional data clustering is a difficult problem in clustering analysis, and solving the clustering problem with high-dimensional data is difficult for the traditional clustering algorithm. For example, there are large numbers of invalid clustering features in high-dimensional sample spaces, and the Euclidean distance is used as a distance measure in the FCM algorithm [13], but it cannot take into account the correlation of each feature space in high-dimensional space. At present, the problem of high-dimensional data is mainly dealt with using feature transformations and feature selection. The method based on feature selection can effectively reduce the dimension and has been widely applied. A subspace-based clustering image segmentation method is proposed in the literature. By defining search strategies and evaluation criteria, effective features for clustering are screened. The original data sets are clustered in different subspaces to reduce storage and computation costs [14].

The existing supervised feature selection method achieves the goal of dimensionality but reduces the operational efficiency. To achieve clustering segmentation using adaptive feature selection, a similarity measurement method for high-dimensional data, which takes into account the correlation between high-dimensional spatial features and effectively reduces the impact of a “dimensional disaster” on high-dimensional data, is proposed in the literature. However, there is a lack of theoretical guidance on how to select the similarity measurement criteria. To avoid any combination search and to apply the method to unsupervised learning, the concept of feature saliency is proposed in the literature. Considering the influence of different features on the clustering results, the Gaussian mixture model is used for clustering analysis to improve the performance of the algorithm [15].

The fuzzy Gaussian mixture models (FGMMs) algorithm replaces the Euclidean distance of the FCM algorithm with the Gaussian mixture model, which can more accurately fit multipeak data and achieves better segmentation of noiseless complex images. Traditional fuzzy C-means clustering analysis treats the different features of samples equally and ignores the important influence of key features on clustering results, which leads to the difference between the clustering segmentation results and the real classification results. According to the theory of feature selection, the concept of feature saliency is used to assume that the saliency of sample features obeys a probability distribution, and the clustering analysis is carried out by using the Gaussian mixture model. Ju and Liu [16] proposed an online feature selection method based on fuzzy clustering, along with its application (OFSBFCM), and a fuzzy C-means clustering method combined with a Gaussian mixture model with feature selection using Kullback–Leibler (KL) divergence (FSFCM) is proposed in this paper [16,17].

In short, the advantages of the feature-based selection of the GMM-based fuzzy clustering algorithm are as follows:

(1) By using the Gaussian mixture model as a distance measure and by accurately fitting multifront data, compared with the FCM algorithm, the FCM algorithm can manage complexly structured data sample sets.
(2) The Gaussian hybrid model algorithm for feature selection assumes that different features of samples play different roles in pattern analysis. Some features play a decisive role in model analysis and overcome the limitations of the FCM algorithm.
  The algorithm treats the different features of samples equally for clustering analysis, ignoring the important influence of key features on the clustering results, which leads to a certain gap between the clustering results and the real classification results.
(3) KL divergence regularization clustering can be widely used in the clustering analysis of class unbalanced data.

The problems of the feature-based GMM-based fuzzy clustering algorithm are as follows:

(1) The parameters need to be adjusted to increase the running time of the algorithm.
(2) Like the FCM algorithm, it only clusters a single pixel without considering the influence of spatial neighborhood pixels on each central pixel. For different types of noisy images, the algorithm does not have good robustness against noise.

## 2. Algorithm Analysis

### 2.1. FLICM Algorithm

The FCM algorithm uses the fuzzy membership degree and nonsimilarity measure to construct the objective function; it also finds the corresponding membership degree and clustering center when the objective function is the smallest in the iteration process to realize the sample classification. Its structure is simple and easy to simulate, and the convergence is fast. However, it does not consider the interference from neighborhood information on the central pixel, and the results of the image segmentation with noise interference are unsatisfactory. To improve the robustness against the noise of the algorithm, Chens et al. [17]. proposed the neighborhood mean and neighborhood mean fuzzy C-means algorithms FCM-S1 and FCM-S2. Later, the Greek scholars Krinidis et al. [18,19]. proposed a neighborhood local fuzzy C-means segmentation algorithm (FLICM), which combines neighborhood pixel spatial information, gray information, and fuzzy classification information to improve the anti-noise performance of the algorithm. Its objective function expression is as follows [20,21]:

(1) $$J={\sum_{i=1}^{N}\sum_{j=1}^{C}z_{ij}^{m}[\left\|x_{i}-v_{j}\right\|}^{2}+G_{ij}]{}^{\prime }$$

(2) $$G_{ij}=\sum_{\beta \in N_{i}}\frac{1}{\tilde{d_{i\beta }}}{\left(1-z_{\beta j}\right)}^{m}d^{2}(x_{\beta },v_{j}).$$

Specifically, $x_{i}={\left(x_{i1},x_{i2},x_{i3},x_{i4},\ldots ,x_{iD}\right)}^{T}$ is the first sample. $x_{i1},x_{i2},x_{i3},x_{i4},\ldots ,x_{iD}$ represent the different attributes of the first sample. *C* is the number of clusters. $z_{ij}$ denotes the fuzzy membership of the first pixel in the *j*th category; the clustering center is $v_{j}(j=1,2,\ldots ,C)$. ${\tilde{d}}_{i\beta }$ is the Euclidean distance of the spatial position between the pixel point and the neighboring pixel $x_{\beta }$. $N_{i}$ represents a set of neighborhood spatial pixels $x_{\beta }$ of pixel point $x_{i}$; the neighborhood window size is 3×3 or 5×5. The optimal iteration expressions of the classification membership degree and the clustering center are as follows [22,23]:

(3) $$z_{ij}=\sum_{k=1}^{c}{\left[\frac{{\left\|x_{i}-v_{j}\right\|}^{2}+G_{ij}}{{\left\|x_{i}-v_{j}\right\|}^{2}+G_{ik}}\right]}^{\frac{-1}{m-1}},$$

(4) $$v_{j}=\frac{\sum_{i=1}^{N}z_{ij}^{m}x_{i}}{\sum_{i=1}^{N}z_{ij}^{m}}.$$

### 2.2. Local Neighborhood Robust Fuzzy Clustering Image Segmentation Algorithm Based on an Adaptive Feature Selection Gaussian Mixture Model

#### 2.2.1. Improved FLICM Algorithm

The FLICM algorithm does not strictly follow the Lagrangian multiplier method to solve the optimal expression of the objective function. Furthermore, it runs too long and falls into local minima. To solve these problems, the unconstrained expression of the objective function is solved using the Lagrangian multiplier method as follows [24,25]:

(5) $$J_{M}={\sum_{i=1}^{N}\sum_{j=1}^{C}z_{ij}^{m}[\left\|x_{i}-v_{j}\right\|}^{2}+G_{ij}]+\sum_{i=1}^{N}\lambda_{i}(1-\sum_{i=1}^{C}z_{ij}).$$

The partial derivative of $J_{M}$ with respect to the membership degree $z_{ij}$ and clustering center $v_{j}$ is obtained, and its partial derivative is 0:

(6) $$\frac{\partial J_{M}}{\partial z_{ij}}=mz_{ij}^{m-1}[{\left\|x_{i}-v_{j}\right\|}^{2}+G_{ij}]-\lambda_{i}=0,$$

(7) $$\frac{\partial J_{M}}{\partial v_{j}}=\sum_{i=1}^{N}z_{ij}^{m}[-2(x_{i}-v_{j})-\sum_{\begin{array}{l} \beta \in N_{i}\\ \beta \neq i \end{array}}\frac{2(x_{\beta }-v_{j}){\left(1-z_{\beta j}\right)}^{m}}{\tilde{d_{i\beta }}+1}]=0.$$

By solving Equations (6) and (7), the following solution is obtained:

(8) $$z_{ij}={\sum_{i=1}^{N}[\frac{{\left\|x_{i}-v_{j}\right\|}^{2}+G_{ij}}{{\left\|x_{i}-v_{k}\right\|}^{2}+G_{ik}}}^{\frac{-1}{\left(m-1\right)}},$$

(9) $$v_{j}=\sum_{k=1}^{C}[\frac{\left\|x_{i}+\sum_{\beta \in N_{i},\beta \neq i}{\left(\tilde{d_{i\beta }}+1\right)}^{-1}(1-z_{\beta j})x_{\beta }\right\|}{\sum_{i=1}^{N}z_{ij}^{m}(1+\sum_{\beta \in N_{i},\beta \neq i}{\left(\tilde{d_{i\beta }}+1\right)}^{-1}{\left(1-z_{\beta j}\right)}^{m})}.$$

Compared with the iteration expressions in the literature, the iteration formula of the clustering centers needs to consider the central pixel $x_{i}$ values. Furthermore, the influence of the neighborhood pixels $x_{\beta }$ on the clustering center $v_{j}$ and the degree of classification membership also have some influence on the clustering center $v_{j}$. To accurately compare the influence of the neighborhood pixels on the central pixels, this section describes the use of neighborhood spatial classification membership $z_{\beta j}$ to restrict the Euclidean distance $d_{i\beta }$ of the spatial position between pixel $x_{i}$ and pixel $x_{\beta }$, and redefines the ambiguity factor $G_{ij}$ to be [26,27]:

(10) $$G_{ij}=\sum_{\beta =N_{1}}\frac{1}{z_{\beta j}\tilde{d_{i\beta }}+1}{\left(1-z_{\beta j}\right)}^{m}d^{2}(x_{\beta },v_{j})$$

(11) $$z_{ij}={\sum_{i=1}^{N}[\frac{{\left\|x_{il}-v_{jl}\right\|}^{2}+G_{ij}}{{\left\|x_{il}-v_{kl}\right\|}^{2}+G_{ik}}}^{\frac{-1}{\left(m-1\right)}}$$

(12) $$z_{ij}=\sum_{k=1}^{C}[\frac{\left\|x_{ij}+\sum_{\beta \in N_{i},\beta \neq i}{\left(z_{\beta j}\tilde{d_{i\beta }}+1\right)}^{-1}(1-z_{\beta j})x_{\beta i}\right\|}{\sum_{i=1}^{N}z_{ij}^{m}(1+\sum_{\beta \in N_{i},\beta \neq i}{\left(z_{\beta j}\tilde{d_{i\beta }}+1\right)}^{-1}{\left(1-z_{\beta j}\right)}^{m})}$$

#### 2.2.2. Local Neighborhood Robust Fuzzy Clustering Algorithm Based on an Adaptive Feature Selection Gaussian Mixture Model

The FLICM algorithm introduces neighborhood spatial information into the objective function of the algorithm to enhance the anti-noise performance of the algorithm; however, the algorithm treats the different features of the samples equally for clustering analysis, ignoring the important impact of key features on the clustering results, which results in unsatisfactory segmentation results. In this section, the idea of feature selection is introduced into the improved FLICM algorithm, KL divergence is introduced as a regularization term to realize feature selection constraints, and a new objective function is obtained as follows [28,29]:

(13) $$J=\sum_{i=1}^{N}\sum_{j=1}^{C}z_{ij}(d(x_{i},v_{j})+G_{ij})+\lambda \sum_{i=1}^{N}\sum_{j=1}^{C}z_{ij}\log\frac{z_{ij}}{\pi_{j}}+\gamma \sum_{i=1}^{N}\sum_{j=1}^{C}\sum_{l=1}^{D}z_{ij}(s_{ijl}\log\frac{s_{ijl}}{\rho_{l}}+(1-s_{ijl})\log\frac{1-s_{ijl}}{1-\rho_{l}})$$

Further, $d_{ij}=\sum_{l=1}^{D}(s_{ijl}{\left(x_{il}-\mu_{jl}\right)}^{2}+(1-s_{ijl}){\left(x_{il}-\varepsilon \right)}^{2}$,

(14) $$G_{ij}=\sum_{\beta \in N_{i}}\frac{{\left(1-z_{\beta j}\right)}^{m}}{z_{\beta j}\tilde{d}+1}d_{\beta j},d_{\beta j}=\sum_{l=1}^{D}(s_{\beta jl}{\left(x_{\beta l}-\mu_{jl}\right)}^{2}+(1-s_{\beta jl}){\left(x_{\beta l}-\varepsilon_{l}\right)}^{2})$$

$d_{ij}$ is the weighted Euclidean distance between the first sample and the center $\mu_{ij}$ of class *J*. The Euclidean distance $d_{\beta j}$ is the spatial position between pixel point $x_{i}$ and pixel point $x_{\beta }$. $s_{ijl}$ is the influence degree of the first characteristic attribute $x_{il}$ on the *j*th class in the fist sample. $\varepsilon_{l}$ is the eigenvalue corresponding to the mean of all samples. $\rho_{l}$ is the weight factor of the first dimension feature attribute of the sample. $G_{ij}$ is used as a fuzzy factor.

In the literature, the membership degree has been obtained strictly according to the Lagrange multiplier method after finding an unconstrained solution of the objective function but the clustering center of the formula solution is directly calculated using the traditional fuzzy C-means clustering cluster center expression, which is not strictly obtained via the Lagrange method, resulting in an inconsistency between Equation (4) and the clustering objective function. In this section, the objective function of clustering is optimized strictly using the Lagrange multiplier method, and the iterative optimization expression is solved. The process is as follows [30,31]:

Finding the partial derivatives of object functions with respect to $s_{ijl}$:

$$\frac{\partial L}{\partial s_{ijl}}=z_{ij}[{\left(x_{il}-\mu_{jl}\right)}^{2}+\sum_{\beta \in N_{i}}\frac{1-z_{\beta j}}{z_{\beta j}\tilde{d_{i\beta }}+1}{\left(x_{\beta l}-\varepsilon_{l}\right)}^{2}]+\gamma z_{ij}(s_{ijl}\log\frac{s_{ijl}}{\rho_{l}}-\log\frac{1-s_{ijl}}{1-\rho_{l}}).$$

Let the partial derivative be zero:

(15) $$s_{ijl}=\frac{\rho_{l}\exp(t_{ij}/\gamma )}{1-\rho_{l}+\rho_{l}\exp(t_{ij}/\gamma )}.$$

The unconstrained expression of the objective function obtained using the Lagrange multiplier method is given by $L_{m}=L-\sum_{i=1}^{N}\eta_{i}(\sum_{i=1}^{N}z_{ij}-1)$. Finding partial derivatives of the formula with respect to $z_{ij}$:

(16) $$\frac{\partial }{\partial z_{ij}}L_{m}=d(x_{i},v_{j})+G_{ij}+\lambda z_{ij}\log\frac{z_{ij}}{\pi_{j}}+\lambda +\gamma \sum_{l=1}^{D}z_{ij}(s_{ijl}\log\frac{s_{ijl}}{\rho_{l}}+(1-s_{ijl})\log\frac{1-s_{ijl}}{1-\rho_{l}})-\eta_{i}$$

Bring the local ambiguity factor $G_{ij}$ into the formula and set the formula equal to zero:

(17) $$\lambda \log\frac{z_{ij}}{\pi_{j}}=\sum_{l=1}^{D}\{s_{ijl}[{\left(x_{il}-u_{jl}\right)}^{2}+\sum_{\beta \in N_{i}}\frac{1-z_{\beta j}}{z_{\beta j}\tilde{d_{\beta j}}+1}{\left(x_{\beta l}-\mu_{jl}\right)}^{2}+\gamma (\log\frac{s_{ijl}}{\rho_{l}}]+(1+s_{ijl}){\left[{\left(x_{il}-\varepsilon_{l}\right)}^{2}+\sum_{\beta \in N_{i}}\frac{1-z_{\beta j}}{z_{\beta j}\tilde{d_{\beta j}}+1}(x_{\beta l}-\varepsilon_{l}\right)}^{2}+\gamma \log\frac{1-s_{ijl}}{1-\rho_{l}})]\}-\lambda +\eta_{i}$$

Constraints of membership degree $\sum_{j=1}^{c}z_{ij}=1$.

The iteration expression of the subordinate degree $z_{ij}$ is solved by introducing Equation (15) into Equation (17), as follows.

(18) $$z_{ij}=\frac{\pi_{j}\exp(-\eta_{ij}/\lambda )}{\sum_{k=1}^{c}\pi \exp(-\eta_{ik}/\lambda )}$$

Therefore:

$$\begin{array}{c} \eta_{ij}=\sum_{l=1}^{D}\{\frac{\rho_{l}\exp(t_{ij}/\gamma )}{1-\rho_{l}+\rho_{l}\exp(t_{ij}/\gamma )}[{\left(x_{il}-u_{jl}\right)}^{2}+\sum_{\beta \in N_{i}}\frac{1-z_{\beta j}}{z_{\beta j}\tilde{d_{\beta j}}+1}{\left(x_{\beta l}-\mu_{jl}\right)}^{2}+\gamma (\log\frac{\rho_{l}\exp(t_{ij}/\gamma )}{1-\rho_{l}+\rho_{l}\exp(t_{ij}/\gamma )}]+\frac{1-\rho_{l}}{1-\rho_{l}+\rho_{l}\exp(t_{ij}/\gamma )}[{\left(x_{il}-\varepsilon_{l}\right)}^{2}\\ +\sum_{\beta \in N_{i}}\frac{1-z_{\beta j}}{z_{\beta j}\tilde{d_{\beta j}}+1}{\left(x_{\beta l}-\varepsilon_{l}\right)}^{2}+\gamma \log\frac{1}{1-\rho_{l}+\rho_{l}\exp(t_{ij}/\gamma )})]\} \end{array}$$

Finding the partial derivatives of the object functions with respect to $\mu_{jl}$ gives:

(19) $$\frac{\partial }{\partial \mu_{jl}}=z_{ij}s_{ijl}(x_{il}-\mu_{jl})+z_{ij}\sum_{\beta \in N_{i}}\frac{1-z_{\beta j}}{z_{\beta j}d_{\beta j}+1}s_{ijl}(x_{\beta l}-\mu_{jl}),$$

(20) $$\mu_{jl}=\frac{1}{M_{jl}}\sum_{i=1}^{N}z_{ij}s_{ijl}x_{il}$$

where $M_{jl}=\sum_{i=1}^{N}z_{ij}s_{ijl}x_{il}$.

Finding the partial derivatives of the object functions with respect to $\varepsilon_{l}$ gives:

(21) $$\frac{\partial }{\partial \varepsilon_{l}}=z_{ij}(1-s_{ijl})(x_{il}-\varepsilon_{l})+z_{ij}\sum_{\beta \in N_{i}}\frac{1-z_{\beta j}}{z_{\beta j}\tilde{d_{\beta j}}+1}(1-s_{ijl})(x_{\beta l}-\varepsilon_{l})$$

Let the partial derivative be zero and obtain the expression of $\varepsilon_{l}$ as follows:

(22) $$\varepsilon_{l}=\frac{1}{F_{l}}\sum_{i=1}^{N}\sum_{j=1}^{C}z_{ij}(1-s_{ij})x_{il}$$

(23) $$F_{l}=\frac{1}{F_{l}}\sum_{i=1}^{N}\sum_{j=1}^{C}z_{ij}(1-s_{ijl})$$

For the objective function with respect to $\rho_{l}$, the partial derivative is obtained, and the partial derivative is set to 0. The iterative expression is as follows.

(24) $$\rho_{l}=\frac{1}{N}\sum_{i=1}^{N}\sum_{j=1}^{C}z_{ij}s_{ijl}$$

Using the Lagrange multiplier method, the partial derivative of the objective function with respect to $\pi_{j}$ is set to 0:

(25) $$\frac{\partial }{\partial \pi_{j}}[L-\sum_{i=1}^{N}\eta_{i}(\sum_{j=1}^{C}\pi_{j}-1)]=0$$

The iterative expression of $\pi_{j}$ is obtained from the above formula:

(26) $$\pi_{j}=\frac{1}{N}\sum_{i=1}^{N}z_{ij}$$

#### 2.2.3. Postprocessing Method of the Clustering Membership Degree

To further enhance the robustness against noise, the neighborhood weighting information is added to the membership degree of the iteration expression. Combined with the idea of the non-Markov random field (MRF) space-constrained Gaussian model in the literature, this section constructs a neighborhood weighting function by using the classification membership degree and the postprocessing clustering membership degree. The function considers the corresponding median to be a probability by classifying the membership degree of neighborhood pixels in ascending order, which is expressed as follows [32,33]:

(27) $$H_{ij}=median\{z_{\beta j}\}$$

A indicates that the neighborhood window sizes are 3×3, 5×5 for the classification membership of neighborhood pixels. $N_{i}$ represents the set of classified membership degrees of neighborhood pixels. According to the Bayesian theorem, the weight factor of the neighborhood information function is added to Equation (18), and the new expression of the membership degree is given in Equation (27):

(28) $$z_{ij}=\frac{\pi_{j}{\left(H_{ij}\right)}^{\alpha }\exp(-\eta_{ij}/\lambda )}{\sum_{k=1}^{C}\pi_{k}{\left(H_{ik}\right)}^{\alpha }\exp(-\eta_{ik}/\lambda )}$$

In this equation, α is the weight factor and the selection range is. a value of 2.0 is usually chosen. Its function is similar to the fuzzy weight factor *m* in the traditional fuzzy C-means clustering objective function.

The improved membership degree of the sample classification in this chapter has the following properties [34,35]:

(1) Neighborhood weighted membership still satisfies the constraints $\sum_{i=1}^{C}z_{ij}=1$.
(2) The membership degree of the current pixel $x_{i}$ in class *J* is proportional to the probability that the neighborhood pixel $x_{\beta }$ belongs to class *J*.

As the probability increases, the degree of membership increases. Conversely, when neighborhood pixel $x_{i}$ belongs to class *j*, the probability tends to zero, and thus, the membership degree of the current pixel $x_{i}$ in class *j* decreases.

In addition, $\varphi_{ij}={\left(H_{ij}\right)}^{\alpha }$, such that:

(29) $$z_{ij}=\frac{\pi_{j}\varphi_{ij}\exp(-\eta_{ij}/\lambda )}{\sum_{k=1}^{C}\pi_{k}\varphi_{ik}\exp(-\eta_{ik}/\lambda )}$$

The derivative is obtained, as follows:

(30) $$\frac{\partial z_{ij}}{\partial \varphi_{ij}}=\frac{\pi \exp(-\eta_{ij}/\lambda )\sum_{k=1,j\neq k}^{c}\pi \varphi_{ik}\exp(-\eta_{ik}/\lambda )}{(\sum_{k=1}^{C}\pi_{k}\varphi_{ik}\exp(-\eta_{ik}/\lambda ){)}^{2}}≻0$$

It is proved that the weighted neighborhood membership degree can be found using the neighborhood information.

The monotone incremental function of $\varphi_{ij}$, which uses the $\varphi_{ij}$ number to restrict the membership degree of classification, improves the performance of the sample classification to a certain extent and enhances the robustness of the algorithm against noise. To achieve image segmentation, the local fuzzy clustering algorithm based on feature selection in this chapter needs to solve the iterative optimization expression. The detailed steps are as follows [36,37]:

Step 1: Transform the image pixel value into sample eigenvector $x_{i}$, where $x_{i}=(x_{i1},\ldots ,x_{iD})(i=1,2,\ldots ,N)$, *N* is the total number of pixels, and *C* is the number of clusters.

The termination condition threshold is δ, the maximum iteration number is $\tau_{\max}$, the regularization parameter is λ, and the feature selection parameter is γ.

Step 2: Initialize the feature attribute weight coefficients $\rho_{l}=1/D$ and $\pi_{j}=1/C(j=1,\ldots ,C)$ to find the prior probability of sample classification.

Step 3: The central vector of the sample classification class is obtained using FCM clustering, where $\mu_{j}=(\mu_{j1},\ldots ,\mu_{jD})$. Class variance matrix is $\sigma_{j}^{2}=(\sigma_{j1}^{2},\ldots ,\sigma_{jD}^{2})$. Sample eigenvalue mean vector is $\varepsilon =(\varepsilon_{l},\ldots ,\varepsilon_{D})$. Eigenvalue variance matrix is $\nu^{2}=(\nu_{l}^{2},\ldots ,\nu_{D}^{2})$. Given the improved adaptive spatial neighborhood information, in this section, the initial values of the Gaussian mixture fuzzy clustering algorithm are selected as follows: $\mu_{j}(0)$$\sigma_{j}^{2}(0)$ε(0)$\nu^{2}(0)$.

Step 4: Compute the adaptive spatial neighborhood information function $H_{ij}$ using Equation (26).

Step 5: Use Equation (15) to calculate the eigenweight function $s_{ijl}$.

Step 6: Calculate the membership function $z_{ij}$ using Equation (28).

Step 7: Update $\mu_{j},\sigma_{j}^{2},\varepsilon ,\nu^{2},\pi_{j},\rho_{l}$ using Equations (20) to (26).

Step 8: If the number of iterations is $\tau =\tau_{\max}$ or the convergence condition $\{\left|z_{ij}^{\left(\tau +1\right)}-z_{ij}^{\left(\tau \right)}\right|\}≺\delta$ is satisfied, the iteration will stop; otherwise, the iteration returns to step 4.

Step 9: The image pixels are classified and segmented according to the principle of the maximum membership degree using the $z_{ij}$ values obtained when the algorithm’s iterations have been completed.

## 3. Experimental Results and Analysis

**Figure 1:**
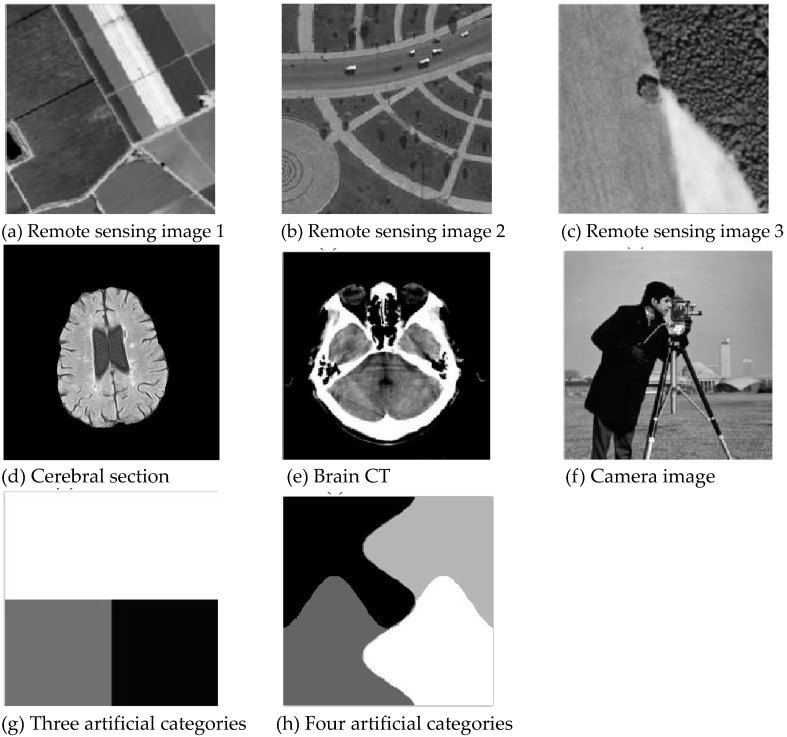
Original images.

To verify the good segmentation performance and anti-noise ability of the improved algorithm, high-resolution remote sensing images, including common ground objects in remote sensing images (such as forest farmland, bare land, and grassland), synthetic images, standard images, and high-resolution medical images were selected, as is shown in Figure 1. The improved algorithm and the FCM-S, FLICM, kernel-weighted FLICM (KWFLICM), and local data and membership relative entropy-based FCM (LDMREFCM) algorithms were used to segment gray images with different noises [36,37]. The peak signal-to-noise ratio (PSNR) and the error misclassification rate (MCR) were used to compare the segmentation performance and anti-noise performance of the algorithms [38,39]. Generally, the MCR is often used to quantitatively evaluate the performance of segmentation algorithms, which is defined as:

(31) $$MCR=[1-(\sum_{j=1}^{C}C_{j}{)}^{-1}\cdot (\sum_{j=1}^{C}A_{j}\cap C_{j})]\times 100\%$$

The efficiency of the algorithms was compared using the running time after convergence and the number of iterations *n*. A Dell OptiPlex 360 (Intel Core 4, 8 GB of memory) running a Windows 7 system with the MATLAB 2013a (MathWorks, Natick, MA, USA)programming environment comprised the evaluation platform. The maximum number of iterations $T_{\max}$ of the algorithm was set to 300. The cluster numbers *C* for each noise was chosen to be 2, 3, and 4. The regularization parameters and characteristic parameters were selected separately to be $\lambda =10^{3}$ and $\gamma =10^{3}$, respectively. The iteration threshold was $\delta =10^{-4}$, and the neighborhood window size was set to 3×3.

### 3.1. Image Segmentation Test with Gaussian Noise

#### 3.1.1. Segmentation Performance Test

**Figure 2:**
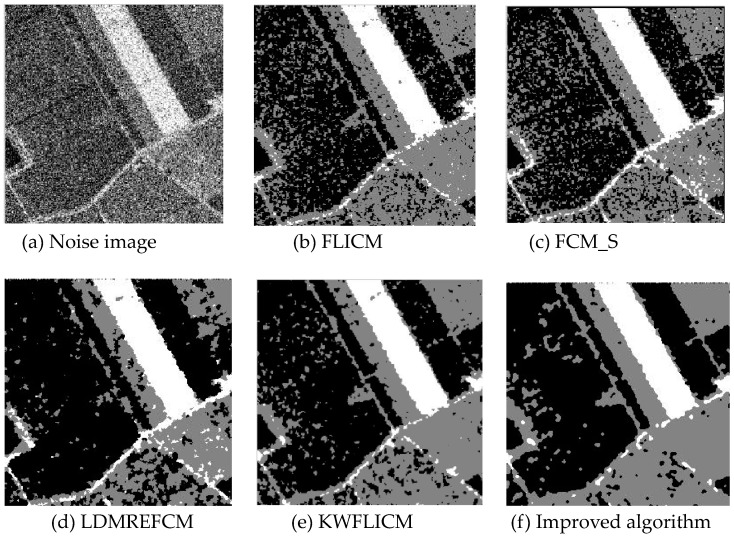
Gaussian noise disturbing remote sensing image 1 (**a**) and the segmentation results (**b**–**f**). FLICM: Fuzzy local information C-means, FCM_S: Fuzzy C-means with spatial constraints, LDMREFCM: Local data and membership relative entropy-based FCM, KWFLICM: Kernel-weighted FLICM, and Improved algorithm.

**Figure 3:**
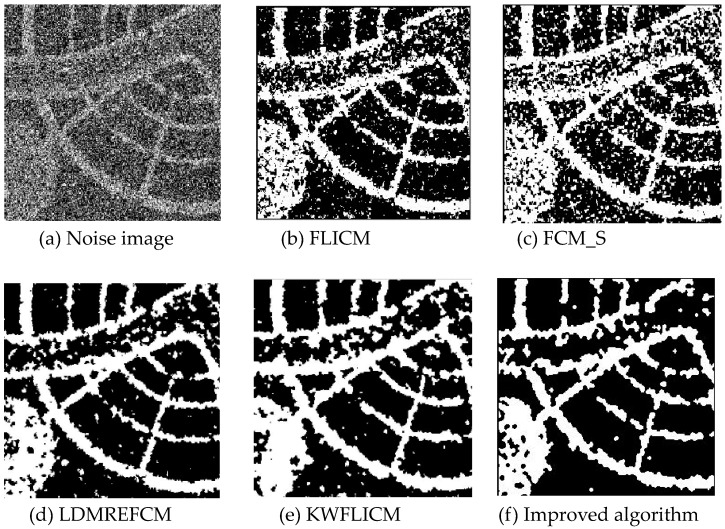
Gaussian noise disturbing remote sensing image 2 (**a**) and the segmentation results (**b**–**f**).

**Figure 4:**
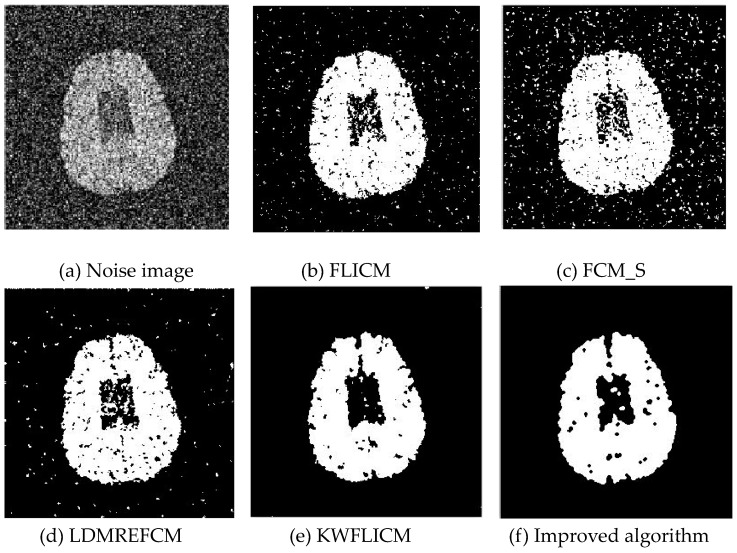
Gaussian noise interfering with the brain slice image (**a**) and the segmentation results (**b**–**f**).

**Figure 5:**
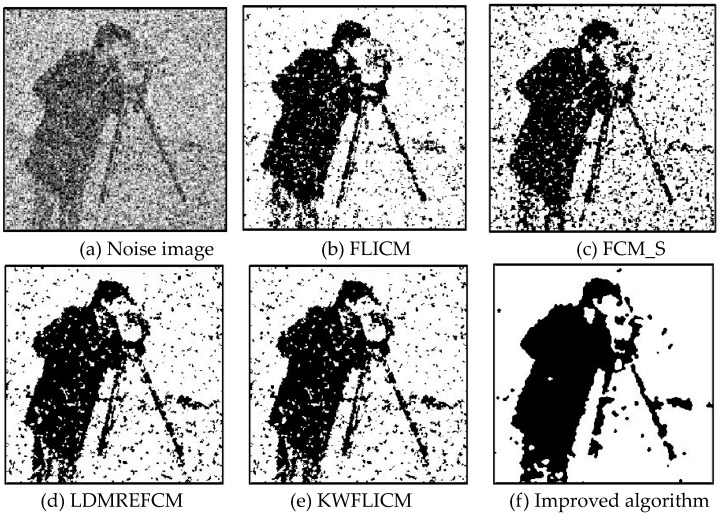
Gaussian noise disturbing the camera image (**a**) and the segmentation results (**b**–**f**).

Gaussian noise was added to two remote sensing images with a mean value of 0 and mean variances of 57 and 80. Gaussian noise was added to images containing four artificial categories, brain CT (Computed Tomography) images, and camera images with a mean value of 0 and mean variances of 140 and 161. The number of clusters was set to 3, 4, 2, and 2. The results were compared using the results from the FLICM, FCM_S, LDMREFCM, and KWFLICM algorithms and the improved algorithm. The original image is shown in Figure 1, and the experimental results are shown in Figure 2, Figure 3, Figure 4 and Figure 5 (b–f). The error rate and PSNR of the segmentation results are shown in Table 1 and Table 2, and the iteration time and the number of iterations are shown in Table 3 [40,41].

#### 3.1.2. Test Result

Comparing the segmentation results of the five algorithms in Figure 2, Figure 3, Figure 4 and Figure 5 for four images with different degrees of Gaussian noise interference, we can see that the segmentation results of the FCM_S, FLICM, and LDMREFCM algorithms still contained many noise points; the KWFLICM algorithm contained fewer noise points; while the improved algorithm has the fewest noise points. Table 1 shows that the improved algorithm had the highest signal-to-noise ratio compared with the other four algorithms, which shows that the improved algorithm had the strongest anti-Gaussian noise ability. Table 2 shows that the segmentation result of the improved algorithm was the smallest of all the algorithms, which shows that the segmentation result of the improved algorithm was closer to the ideal segmentation result and had a better segmentation performance. Comparing the PSNR and iteration time of each algorithm in Table 3, the average PSNR of the improved algorithm was 0.7 dB higher than that of the KWFLICM algorithm, and the average iteration time of the improved algorithm was 500 s less than that of the KWFLICM algorithm [42,43]. The iteration times of the FCM_S and FLICM algorithms were the lowest, but the difference between the improved algorithm results and the PSNR was 2–5 dB. The anti-noise ability of the FLCM and FCM_S method was poor. Combining the PSNR test results and the iteration time, the improved algorithm had a better anti-Gaussian noise segmentation performance.

### 3.2. Image Segmentation Test of Salt-and-Pepper Noise

#### 3.2.1. Segmentation Performance Test

**Figure 6:**
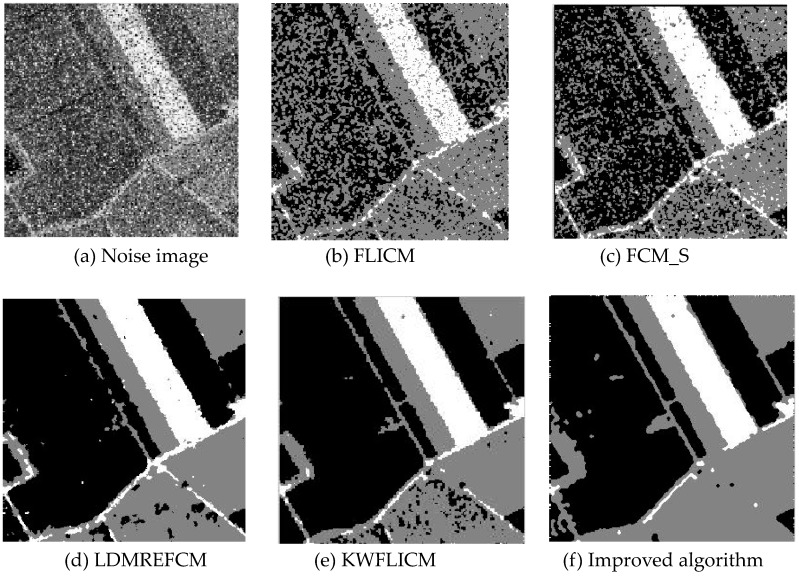
Disturbance of salt-and-pepper noise on remote sensing image 1 (**a**) and the segmentation results (**b**–**f**).

**Figure 7:**
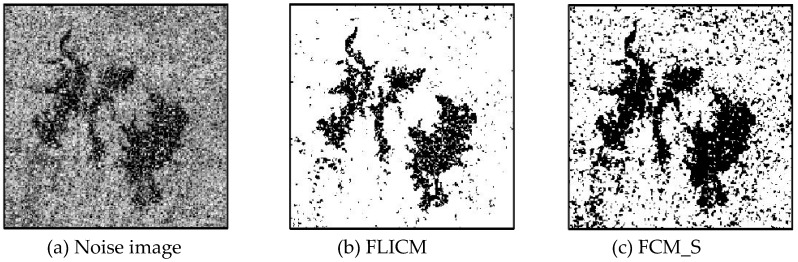

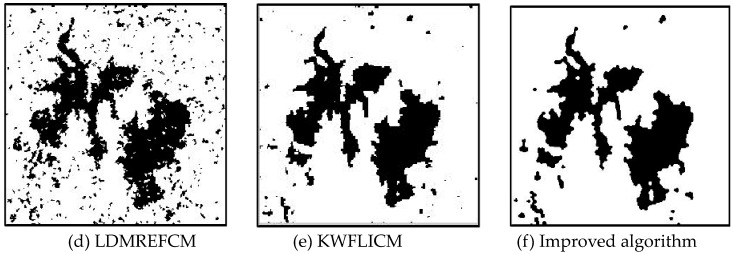
Remote sensing image 4 disturbed using salt-and-pepper noise (**a**) and the segmentation results (**b**–**f**).

**Figure 8:**
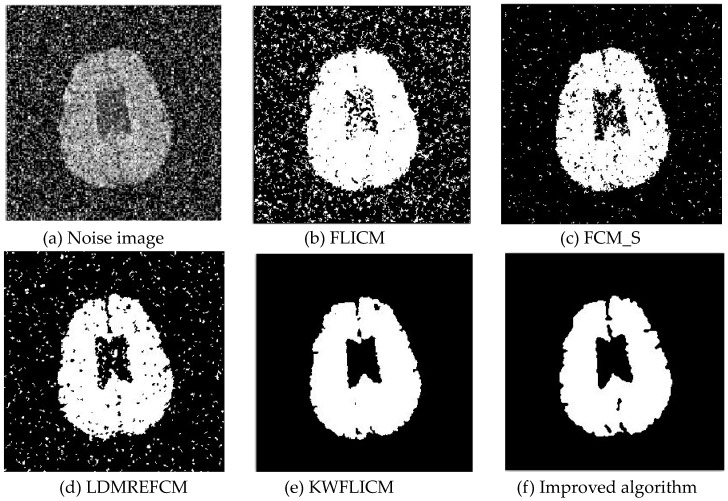
Salt-and-pepper noise interfering with brain slice images (**a**) and the segmentation results (**b**–**f**).

**Figure 9:**
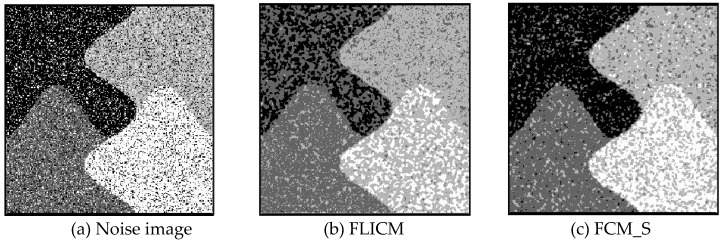

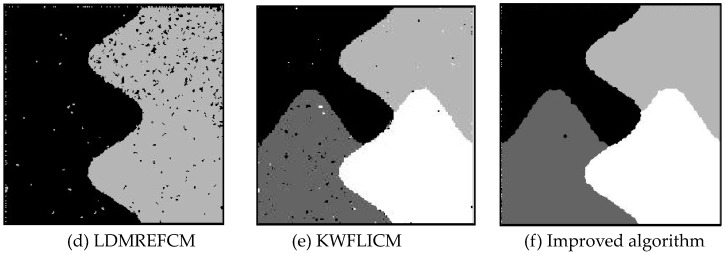
Images containing four artificial categories disturbed by salt-and-pepper noise (**a**) and the segmentation results (**b**–**f**).

In this experiment, 20% and 40% salt-and-pepper noise were added to two remote sensing images, respectively, while 40% and 30% salt-and-pepper noise were added to brain CT images and images containing four artificial categories, respectively. The experimental results are shown in Figure 6, Figure 7, Figure 8 and Figure 9. The number of clusters was set to 3, 4, 2, and 2. The PSNRs and error rates are shown in Table 4 and Table 5, respectively, and the iterative operation time and number of iterations are shown in Table 6.

#### 3.2.2. Test Result

Comparing the results of image segmentation with the multiplicative noise in Figure 6, Figure 7, Figure 8 and Figure 9, we can see that the FCM_S and FLICM algorithms took neighborhood information into account and suppressed some of the multiplicative noise, but in the case of high noise interference, compared with the improved algorithm, the segmentation results contained a large amount of noise. As seen from the results of the artificial segmentation in Figure 6, Figure 7, Figure 8 and Figure 9, the LDMREFCM algorithm produced the phenomenon of false segmentation. The KWFLICM algorithm and the improved algorithm could remove a large number of noise points. From the test results of the PSNR and the error rate (ERR) of the algorithms in Table 4 and Table 5, along with the iteration times of the algorithms in Table 6, it can be concluded that compared with the PSNR of the FCM_S and FLICM algorithms, the LDMREFCM, KWFLICM, and improved algorithms had a significantly greater noise suppression ability. Table 6 shows that the iteration time of the improved algorithm was the lowest. Although the PSNR of the improved algorithm was 0.7 dB less than that of the KWFLICM algorithm [44,45], the iteration time was 300 s less than that of the KWFLICM algorithm, and the PSNR of the brain CT image segmentation test results in Table 6 was 0.7 dB less than that of the KWFLICM algorithm. However, the iteration time was 45 s less than that of the KWFLICM algorithm. In summary, the proposed algorithm showed a superior performance compared with the FCM_S, FLICM, KWFLICM, and LDMREFCM algorithms, where a large amount of salt-and-pepper noise is suppressed, and the iteration speed of the algorithm was faster.

### 3.3. Image Segmentation Test with Multiplicative Noise

#### 3.3.1. Segmentation Performance Test

**Figure 10:**
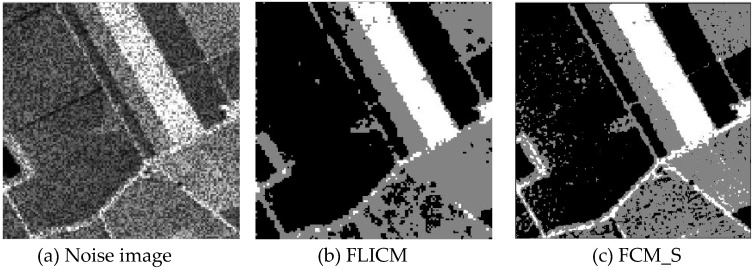

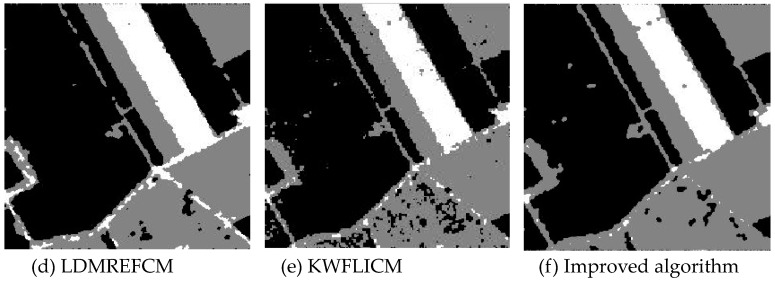
Multiplicative noise disturbing remote sensing image 1 (**a**) and the segmentation results (**b**–**f**).

**Figure 11:**
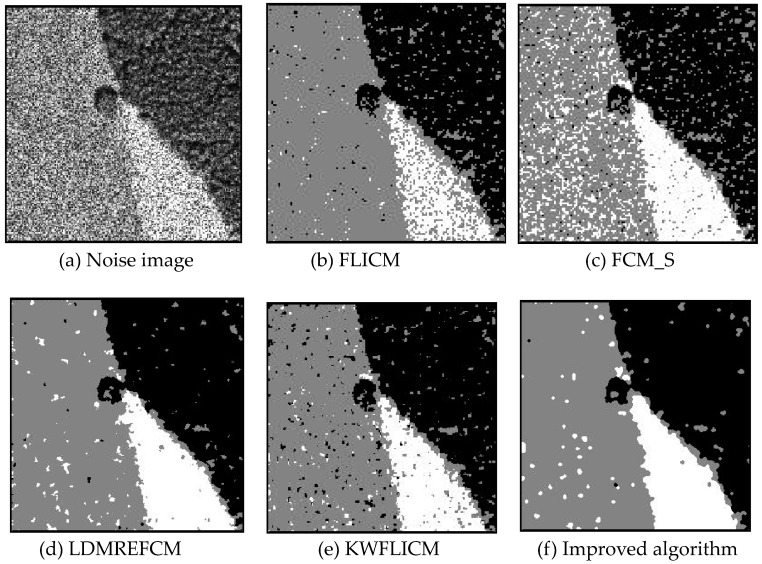
Multiplicative noise disturbing remote sensing image 3 (**a**) and the segmentation results (**b**–**f**).

**Figure 12:**
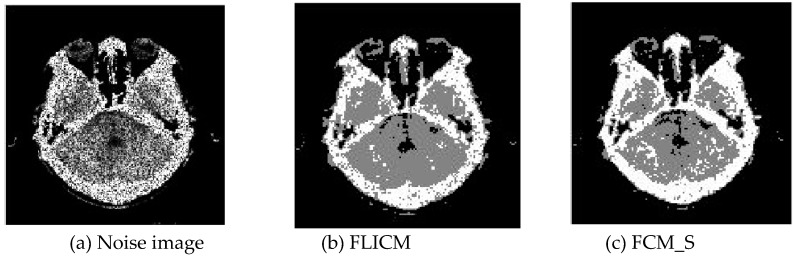

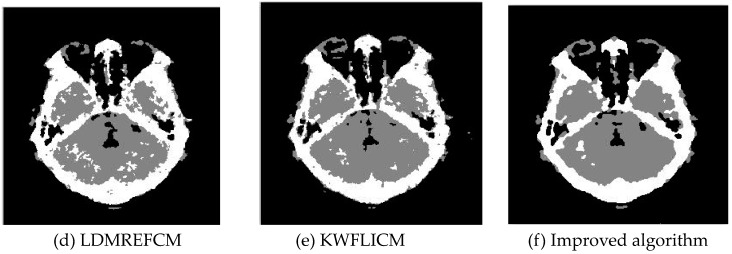
Multiplicative noise disturbing brain CT images (**a**) and the segmentation results (**b**–**f**).

**Figure 13:**
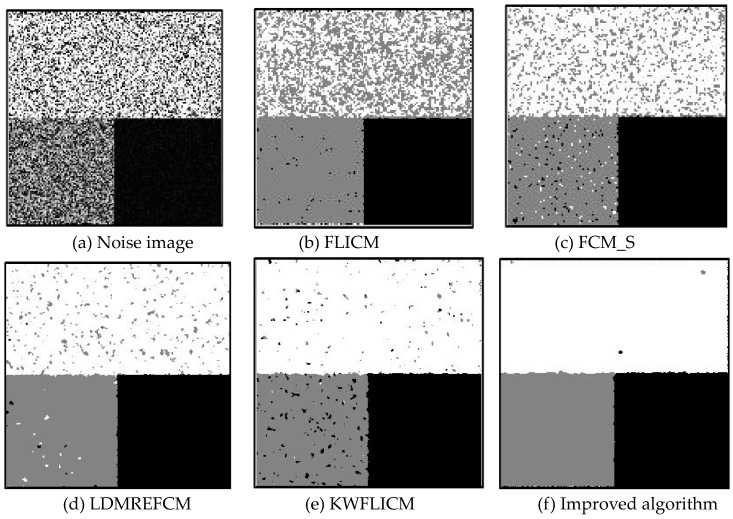
Multiplicative noise interfering with three types of artificial images (**a**) and the segmentation results (**b**–**f**).

Multiplicative noise was added to the remote sensing image, the medical image, and the man-made image with a mean value of 0 and mean variances of 80, 114, 140, and 161. The number of clusters was set to 3, 4, 2, and 2. The experimental results are shown in Figure 10, Figure 11, Figure 12 and Figure 13. The error rate of the segmentation results is shown in Table 7 and Table 8. The iteration times and number of iterations of the algorithms are shown in Table 9 [46,47,48].

#### 3.3.2. Test Result

Comparing the results of the image segmentation with multiplicative noise in Figure 10, Figure 11, Figure 12 and Figure 13, we can see that the FCM_S and FLICM algorithms took neighborhood information into account and suppressed part of the multiplicative noise. The KWFLICM and LDMREFCM algorithms could remove a large number of noise points. Compared with the other algorithms, the improved algorithm contained the fewest noise points. The edges of the segmentation results were continuous and smooth [49]. Compared with Table 7, the PSNR of the improved algorithm was the largest, which proved that the improved algorithm had a better robustness against multiplicative noise. Comparing the error rate of the segmentation results of each algorithm in Table 8 shows that the segmentation results of this algorithm were closer to the ideal segmentation results and had a better segmentation performance. Combined with the comparison of the iteration times in Table 9, the segmentation performance and PSNR of the KWFLICM algorithm were lower than those of the improved algorithm, and the iteration time of the improved algorithm was much shorter than that of the KWFLICM algorithm. In conclusion, the improved algorithm not only guaranteed good robustness against noise, but also reduced the iteration time and improved the operation efficiency of the algorithm.

### 3.4. Segmentation Performance Test

**Figure 14:**
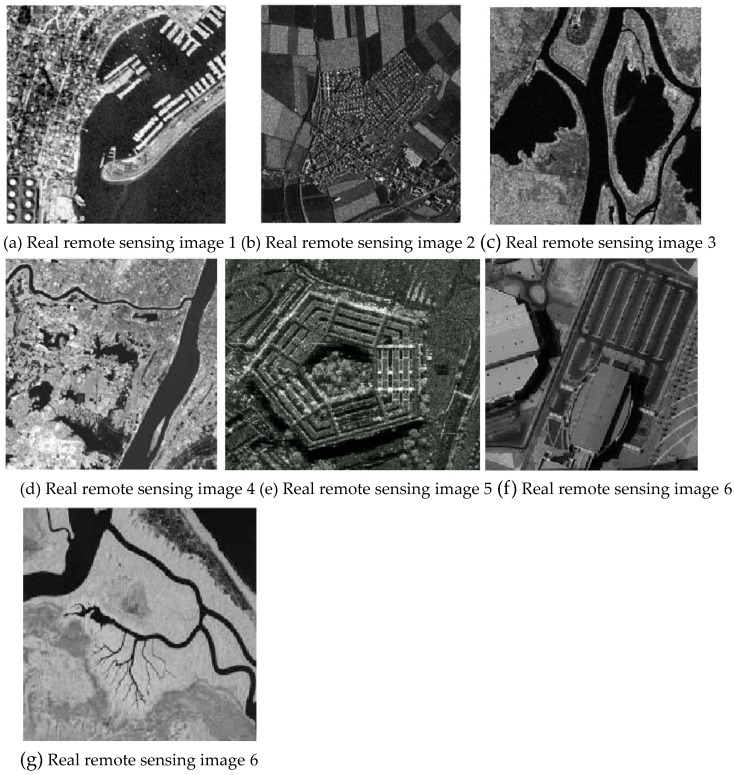
Real remote sensing images.

To test the segmentation efficiency of the algorithm, several real remote sensing images of different sizes were selected for segmentation. Table 10 shows the segmentation time comparison of the five real remote sensing images of different sizes (Figure 14a–g, with sizes of 256 × 256, 532 × 486, 350 × 290, 500 × 500, 590 × 490, 700 × 680, 1024 × 768, respectively), among which, the bold value is the optimal value. It can be seen from this that the segmentation efficiency of the first four comparison algorithms on each real remote sensing image is lower, and the larger the image scale is, the longer the segmentation time is; the improved algorithm can achieve less segmentation time for real remote sensing images of different sizes, and the segmentation efficiency is much higher than other algorithms. The above analysis shows that the algorithm proposed in this paper has high efficiency, and it has certain practical significance and reference value for large-scale remote sensing image processing in practical applications.

### 3.5. Segmentation Test of Remote Sensing Images Disturbed Using Mixed Noise

**Figure 15:**
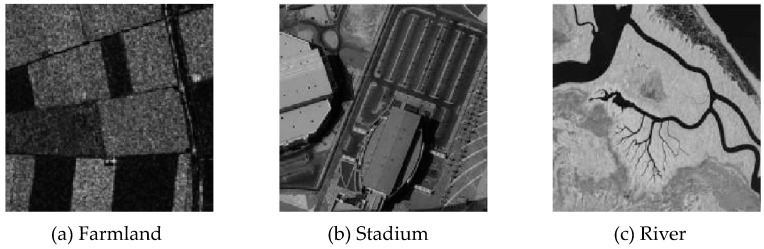
Original remote sensing images.

**Figure 16:**
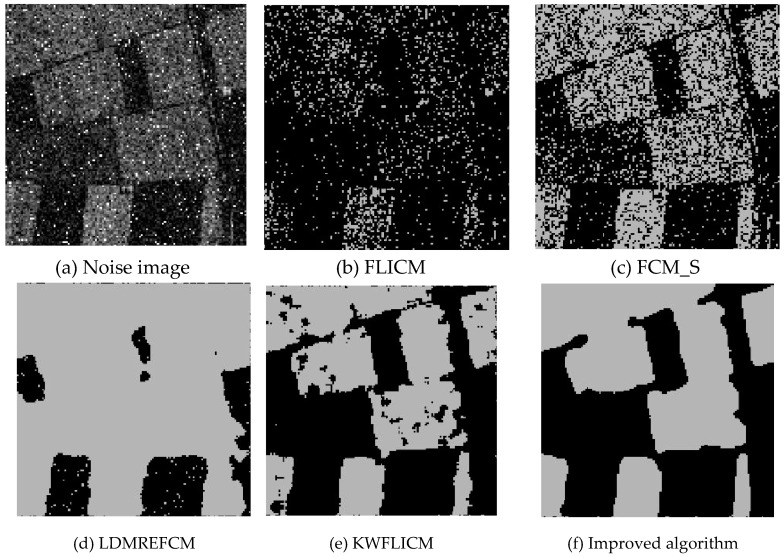
Interference of mixed noise on the farmland image (**a**) and the segmentation results (**b**–**f**).

**Figure 17:**
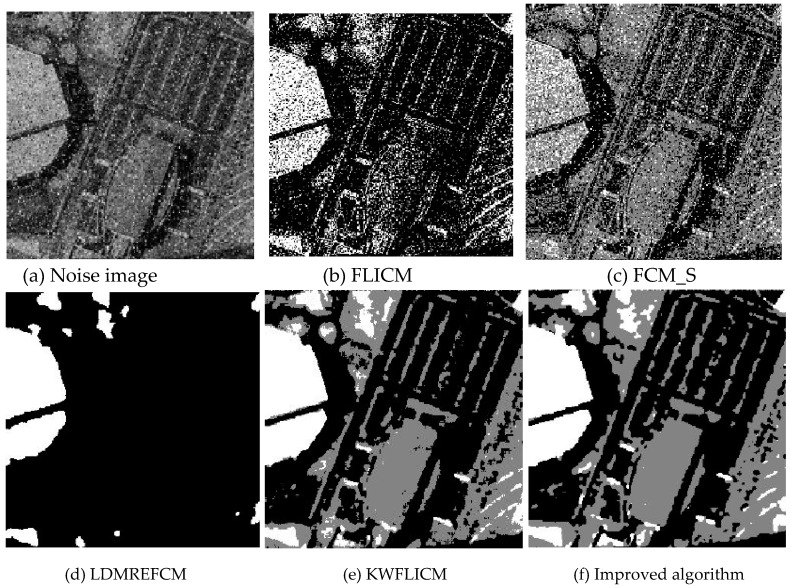
Interference of mixed noise on the stadium image (**a**) and the segmentation results (**b**–**f**).

**Figure 18:**
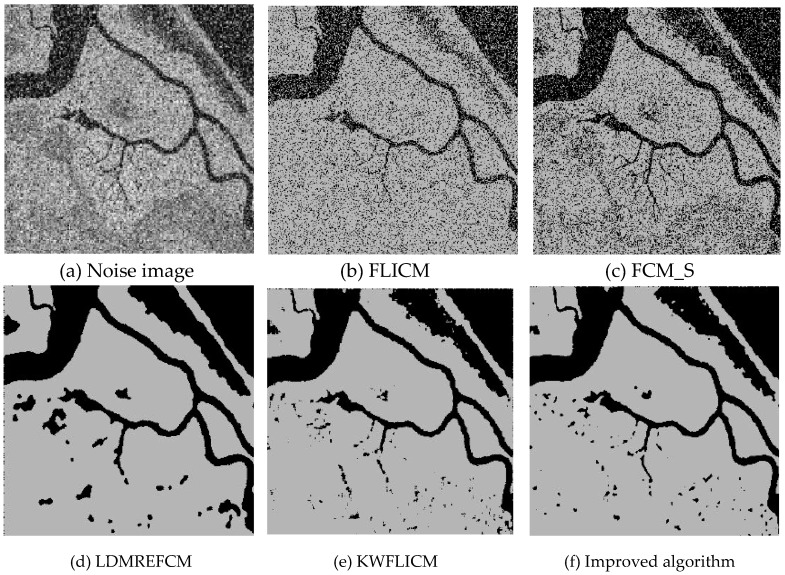
Interference of mixed noise on the river image (**a**) and the segmentation results (**b**–**f**).

Three remote sensing images, including farmland, a stadium, and a river (Figure 15), were segmented and tested by adding Gaussian noise (mean value was 0, mean square deviation was 25) and salt-and-pepper noise of different intensities (5%, 10%, and 30%). The number of clusters was set to 2, 3, and 2, and the segmentation results are shown in Figure 16, Figure 17 and Figure 18.

Compared with the other five algorithms, the improved algorithm was more suitable for the needs of image segmentation disturbed by salt-and-pepper and Gaussian mixture noise, as is shown in Table 11 and Table 12.

## 4. Conclusions

The FLICM algorithm combines neighborhood pixel spatial information, gray information, and fuzzy classification information, which improves the anti-noise performance of the algorithm. However, the algorithm does not take into account the impact of different features on clustering. Additionally, the FLICM algorithm does not minimize the objective function strictly according to the Lagrange method, it easily falls into local optima, and the iteration speed is slow. In this study, the FLICM algorithm was improved. First, the membership degree was introduced into the local constraint information of the FLICM algorithm. Considering the influence of features on clustering, the feature saliency was then introduced into the objective function of the algorithm. Finally, the neighborhood weighting function was constructed using the classification membership degree, and the membership degree was processed to obtain the feature-based membership. The local fuzzy clustering algorithm was selected. The improved algorithm was compared with the existing robust clustering segmentation algorithm in a clustering segmentation test of noisy images. The segmentation results were objectively compared based on the PSNR and error rate, which proved the effectiveness and practicability of the proposed algorithm.

## Figures and Tables

**Table 1:** Comparison of the peak signal-to-noise ratio (PSNR) (dB) for the anti-noise and Gaussian noise after using each algorithm.

Split Image	FLICM	FCM_S	LDMREFCM	KWFLICM	Improved Algorithm
Remote sensing image 1 (57)	13.5301	13.0150	14.0443	15.4893	15.9516
Remote sensing image 2 (80)	8.1045	6.8466	9.3648	8.9022	9.4914
Brain slice image (140)	13.7431	11.6259	13.9768	16.1645	16.2804
Cameraman (161)	9.4264	8.0588	10.0477	12.5087	13.2316

**Table 2:** Comparisons of the misclassification rate (MCR) (%) against Gaussian noise using different algorithms.

Split Image	FLICM	FCM_S	LDMREFCM	KWFLICM	Improved Algorithm
Remote sensing image 1 (57)	17.13	19.60	14.93	11.00	9.84
Remote sensing image 2 (80)	15.47	20.60	11.57	12.88	11.24
Brain slice image (140)	4.22	6.88	4.01	2.42	2.40
Cameraman (161)	12.29	13.07	9.89	5.61	4.75

**Table 3:** Comparison of the iteration times and number of iterations.

Split Image	Iteration Time and Number	FLICM	FCM_S	LDMREFCM	KWFLICM	Improved Algorithm
Remote sensing image 1 (57)	$t_{s}(s)$	26.442	2.855	15.4893	1465.473	760.046
	*n*	47	18	137	57	56
Remote sensing image 2 (80)	$t_{s}(s)$	13.728	1.014	189.209	1265.792	226.326
	*n*	40	16	56	88	28
Brain slice image (140)	$t_{s}(s)$	8.284	1.498	60.017	248.337	180.134
	*n*	22	17	2	15	19
Cameraman (161)	$t_{s}(s)$	15.179	1.389	181.138	226.937	180.611
	*n*	41	16	51	13	19

**Table 4:** Comparison of the PSNR (dB) for algorithms applied to images disturbed by salt-and-pepper noise.

Split Image	FLICM	FCM_S	LDMREFCM	KWFLICM	Improved Algorithm
Remote sensing image 1 (20%)	10.9083	12.1014	17.7271	19.3663	18.5909
Remote sensing image 2 (40%)	10.9748	8.1522	10.3905	13.2863	15.5261
Cerebral section (40%)	9.1041	8.8360	12.6613	18.6563	17.9488
Four artificial categories (30%)	13.3005	15.2344	11.3057	25.4605	25.6753

**Table 5:** Comparison of the MCR (%) for algorithms applied to images disturbed by salt-and-pepper noise.

Split Image	FLICM	FCM_S	LDMREFCM	KWFLICM	Improved Algorithm
Remote sensing image 1 (20%)	31.21	23.37	6.11	4.50	5.22
Remote sensing image 2 (40%)	7.99	15.30	9.14	4.69	2.80
Cerebral section (40%)	12.29	13.07	5.42	1.36	2.38
Four artificial categories (30%)	38.57	23.36	52.48	1.48	1.43

**Table 6:** Operation time and number of iterations for each algorithm.

Split Image	Iteration Time and Numbe	FLICM	FCM_S	LDMREFCM	KWFLICM	Improved Algorithm
Remote sensing image 1 (20%)	$t_{s}(s)$	10.582	2.933	494.152	620.837	332.492
	*n*	20	27	97	24	23
Remote sensing image 2 (40%)	$t_{s}(s)$	18.549	4.227	165.631	224.407	252.513
	*n*	50	15	45	14	26
Cerebral section (40%)	$t_{s}(s)$	8.58	1.482	213.65	230.743	185.451
	*n*	23	17	13	13	17
Four artificial categories (30%)	$t_{s}(s)$	21.481	4.337	580.81	179.561	123.545
	*n*	31	33	78	6	6

**Table 7:** Comparison of the PSNR (dB) for the multiplicative noise resistance of algorithms.

Split Image	FLICM	FCM_S	LDMREFCM	KWFLICM	Improved Algorithm
Remote sensing image 1 (80)	17.1948	16.4000	18.5557	17.7491	18.2079
Remote sensing image 3 (114)	16.2157	16.9107	18.8884	17.2875	19.3224
Brain CT (140)	17.2170	17.4349	18.3648	19.2218	19.2364
Artificial, three categories (161)	12.0180	14.5374	20.1556	20.8528	24.1451

**Table 8:** Comparison of the MCR (%) for multiplicative noise resistance of algorithms.

Split Image	FLICM	FCM_S	LDMREFCM	KWFLICM	Improved Algorithm
Remote sensing image 1 (80)	7.48	8.63	5.49	6.62	6.00
Remote sensing image 3 (114)	9.51	8.72	5.09	7.27	4.61
Brain CT (140)	7.60	7.15	3.78	4.43	1.36
Artificial, three categories (161)	25.65	12.06	3.67	2.19	0.81

**Table 9:** Iteration time and number of iterations for each algorithm.

Split Image	Iteration Time and Numbe	FLICM	FCM_S	LDMREFCM	KWFLICM	Improved Algorithm
Remote sensing image 1 (80)	$t_{s}(s)$	26.395	2.839	352.08	326.783	341.965
	*n*	50	26	66	14	25
Remote sensing image 3 (114)	$t_{s}(s)$	17.721	3.495	580.462	1225.57	407.895
	*n*	33	32	105	49	30
Brain CT (140)	$t_{s}(s)$	17.666	1.841	308.691	541.184	407.535
	*n*	33	17	60	24	30
Artificial, three categories (161)	$t_{s}(s)$	22.278	1.076	261.068	1116.625	198.809
	*n*	41	10	49	49	14

**Table 10:** Segmentation time of real remote sensing images with different sizes by each algorithm.

Segmentation Algorithm	Figure 14a	Figure 14b	Figure 14c	Figure 14d	Figure 14e	Figure 14f	Figure 14g
FCM_S	2.0428	6.2186	2.1626	5.5815	8.6430	10.248	13.976
FLICM	5.1719	20.7453	5.0523	15.4640	22.2379	24.543	26.787
KWFLICM	5.9442	22.9801	7.0612	23.4308	24.1369	26.453	28.285
LDMREFCM	4.5021	20.6175	5.2836	13.4459	17.5801	19.456	22.167
Improved algorithm	**1.1204**	**3.2987**	**1.4128**	**2.2367**	**4.2376**	**6.298**	**8.213**

**Table 11:** The PSNR (dB) comparison between different algorithms against mixed noise.

Split Image	FLICM	FCM_S	LDMREFCM	KWFLICM	Improved Algorithm
Farmland	6.3382	8.9837	9.2103	7.7234	15.7468
Stadium	8.3001	9.4640	9.1565	9.5530	15.1158
Rivers	10.0101	10.0697	10.4482	12.4691	17.4963

**Table 12:** The MCR (%) comparison against anti-mixed noise between each algorithm.

Split Image	FLICM	FCM_S	LDMREFCM	KWFLICM	Improved Algorithm
Farmland	46.44	25.34	37.14	24.07	5.34
Stadium	44.82	34.90	41.19	33.99	10.93
Rivers	20.02	19.75	11.37	18.01	3.57
